# Annual—Universal Medical—Science

**Published:** 1890-04

**Authors:** 


### Book Notices

Annual of the Universal Medical Sciences. A Yearly Report of the Progress of the General Sanitary Sciences throughout the World. Edited by Chas. E. Sajous, M. D., Lecturer on Laryngology and Rhinology in Jefferson Medical College, Philadelphia, etc., and Seventy Associated Editors; Assisted by Over Two Hundred Corresponding Editors, Collaborators and Correspondents. Illustrated with Chromo-lithographs, Engravings and Maps, 5 Vols. F. A. Davis, Philadelphia, New York and London, 1889.

On the first issue of the Annual, that is the edition of 1888, the writer considered the work one of the greatest value and almost perfect in the arrangement of subject matter, index, and general style, but the edition of 1889 is a decided improvement in many respects over the first edition. For instance a complete index is found in each volume. Both systems of weights and measures are given side by side. The plan of giving proper credit to journals has also been improved upon. The work altogether is a marvel of beauty and excellence. It is intended to include everything that is new or worthy, culled from all scientific publications during the year the world over. If you have written anything that deserves to live look for it in the Annual with proper credit. The best of everything is noticed. The rubbish is left behind. A comprehensive grasp of the whole field can be had by the perusal of these volumes. They are cheaper than the bound journals, because they include every tongue and clime and the dross is left out. B.

